# Isobutene production in *Synechocystis* sp. PCC 6803 by introducing α-ketoisocaproate dioxygenase from *Rattus norvegicus*

**DOI:** 10.1016/j.mec.2021.e00163

**Published:** 2021-01-23

**Authors:** Henna Mustila, Amit Kugler, Karin Stensjö

**Affiliations:** Microbial Chemistry, Department of Chemistry-Ångström Laboratory, Uppsala University, SE-751 20, Uppsala, Sweden

**Keywords:** Cyanobacteria, *Synechocystis*, Metabolic engineering, Isobutene production, α-ketoisocaproate dioxygenase, Mevalonate-3-kinase, HDC, High density cultivation, HMB, β-hydroxy-β-methylbutyrate, HPP, 4-hydroxyphenylpyruvate, KIC, α-ketoisocaproate, KICD, α-ketoisocaproate dioxygenase, M3K, Mevalonate-3-kinase, OD_750_, Optical density at 750 ​nm

## Abstract

Cyanobacteria can be utilized as a platform for direct phototrophic conversion of CO_2_ to produce several types of carbon-neutral biofuels. One promising compound to be produced photobiologically in cyanobacteria is isobutene. As a volatile compound, isobutene will quickly escape the cells without building up to toxic levels in growth medium or get caught in the membranes. Unlike liquid biofuels, gaseous isobutene may be collected from the headspace and thus avoid the costly extraction of a chemical from culture medium or from cells. Here we investigate a putative synthetic pathway for isobutene production suitable for a photoautotrophic host. First, we expressed α-ketoisocaproate dioxygenase from *Rattus norvegicus* (*Rn*KICD) in *Escherichia coli*. We discovered isobutene formation with the purified *Rn*KICD with the rate of 104.6 ​± ​9 ​ng (mg protein)^-1^ min^-1^ using α-ketoisocaproate as a substrate. We further demonstrate isobutene production in the cyanobacterium *Synechocystis* sp. PCC 6803 by introducing the *Rn*KICD enzyme. *Synechocystis* strain heterologously expressing the *Rn*KICD produced 91 ​ng ​l^−1^ OD_750_^−1^ ​h^−1^. Thus, we demonstrate a novel sustainable platform for cyanobacterial production of an important building block chemical, isobutene. These results indicate that *Rn*KICD can be used to further optimize the synthetic isobutene pathway by protein and metabolic engineering efforts.

## Highlights

• Photosynthetic isobutene production is demonstrated in a cyanobacterium.
• A *Synechocystis* strain capable of continuous direct conversion of CO_2_ to isobutene.
• α-ketoisocaproate dioxygenase from *R. norvegicus* (*Rn*KICD) is determined to form isobutene.
• *Rn*KICD can convert α-ketoisocaproate to isobutene both *in vitro* and *in vivo*.

## Graphical abstract

**Figure:**
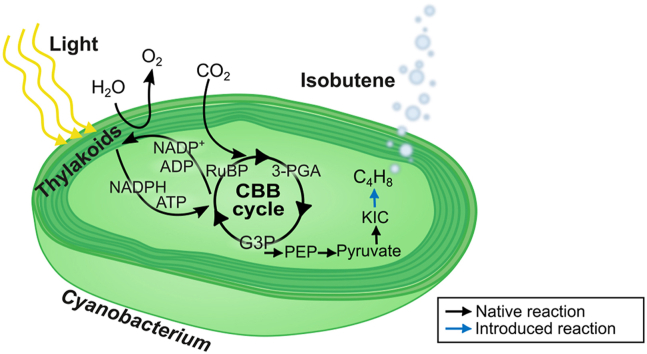

## 1 Introduction

Isobutene (2-Methylpropene) is a small volatile and highly reactive alkene. As a platform chemical, isobutene is an important starting material for the production of alkylate and polymer gasoline, butyl rubber and speciality chemicals (Geilen et al., 2014). Oligomerization and hydrogenation of isobutene can also be used for production of kerosene type jet-fuels (Nicholas 2017). Annual production of isobutene is approximately 15 million tons with a market value exceeding USD 20 billion (Grand View Research, 2016). Currently, isobutene is almost exclusively produced from fossil sources through petrochemical cracking of crude oil. As we are facing a global climate crisis, there is an urgent need for renewable alternatives for crude oil-based products. Attempts to produce isobutene from renewable sources have focused on two approaches: (i) production of bio-based isobutanol via bacterial fermentation followed by dehydration of isobutanol to isobutene using metal catalysts (Taylor et al., 2010); (ii) introducing a complete artificial metabolic pathway to convert glucose to isobutene by bacterial fermentation (van Leeuwen et al., 2012). Both of the described bio-isobutene routes start with biomass-derived sugars and are therefore limited by the availability of sustainably produced biomass. Moreover, it was estimated that more than 70% of the costs for isobutene production at commercial scale are due to the utilization of fermentable sugars as the feedstock for the heterotrophic micro-organisms. (van Leeuwen et al., 2012).

For biological isobutene production, the key is to identify enzymes and pathways for isobutene production. Currently, four types of enzymes are known to produce isobutene. A cytochrome P450 (P450, Uniprot no. Q12668) isolated from microsomes of the yeast in *Cystobasidium minutum* (*Rhodotorula minuta*) (*Cm*P450) was found to generate isobutene using isovalerate as a substrate (Fukuda et al., 1994). The highest production rate reached 41 ​μg ​g^−1^ ​h^−1^ when the yeast culture was supplemented with glucose, L-leucine, L-phenylalanine and oxygen (Fujii et al., 1987). The membrane associated and heme-containing cytochrome P450s are difficult to express in bacterial systems (Hausjell et al., 2018). Further improvement on the isobutene production yield has not been reported since the original studies with the yeast *Cm*P450. A second suggested enzyme is an oleate hydratase that can dehydrate isobutanol to isobutene, however the enzyme variants capable of the reaction are not specified (Marlière 2011a).

The two other known isobutene forming enzymes use β-hydroxy-β-methylbutyrate (HMB, 3-hydroxyisovalerate) as a substrate. Mevalonate diphosphate decarboxylase (*Sc*MDD) from *Saccharomyces cerevisiae* was shown to form isobutene from HMB when heterologously expressed in *E. coli* (Gogerty and Bobik 2010). A genetically modified variant of the *Sc*MDD was able to synthetize isobutene with the rate of 0.33 ​μg ​g^-1^ h^-1^. Another identified enzyme in HMB pathway is mevalonate-3-kinase from *Picrophilus torridus* (*Pt*M3K) that was shown to convert HMB to an unstable 3-phosphoisovalerate intermediate followed by spontaneous decarboxylation to form isobutene (Rossoni et al., 2015). The reported isobutene formation rate with the purified *Pt*M3K was 162 ​ng (mg protein)^-1^ min^-1^. Intact *E. coli* cells expressing the *Pt*M3K were forming isobutene with the rate of 1.7 ​μg ​g^-1^ h^-1^ when cells were provided with 50 ​mM HMB exogenously. The isobutene production rate of *Pt*M3K was approximately five times higher than for *Sc*MDD, yet clearly below commercially feasible rate. Gogerty and Bobik (2010) estimated that minimum 2 ​g ​l^-1^ h^-1^ of isobutene is needed for a feasible commercial fermentation system.

Cyanobacteria are oxygenic photosynthetic micro-organisms that convert CO_2_ directly to sugars and other metabolites. Cyanobacteria can be engineered to produce valuable compounds that they do not necessarily produce naturally. For example, an engineered *Synechocystis* strain was recently shown to channel more than 50% of the assimilated CO_2_ into production of aromatic amino acids (Brey et al., 2020). Unlike heterotrophic production hosts, cyanobacteria are photoautotrophic and not dependent on glucose or other biomass derived feedstocks. Our goal was to identify and design a possible pathway for producing isobutene and introduce it to a model cyanobacterium *Synechocystis* sp. PCC 6803 (hereafter *Synechocystis*) and operate with minimal input, providing water, light, CO_2_ and micronutrients. The *Pt*M3K seemed to be the most prominent enzyme to apply for *Synechocystis*, but a synthetic route for the production of the intermediate HMB needed to be identified. Two pathways for HMB have previously been suggested. One pathway was hypothesized, but not experimentally proven, to start from glucose via acetoacetyl-CoA with several enzymatic steps leading to methylcrotonyl-CoA and subsequently to HMB (Gogerty and Bobik, 2010). Marlière (2011b) described a shorter pathway for enzymatic conversion of acetone and acetyl-CoA into HMB *in vivo* in a European patent application. However, when biosynthesis pathways are introduced in cyanobacteria, titers of chemicals derived from acetyl-CoA are usually much lower than when the pathway branches from pyruvate (Miao et al., 2020). Under photosynthetic conditions, the intracellular pool of acetyl-CoA is less than 5% of that of pyruvate in *Synechocystis* (Dempo et al., 2014). Therefore, we decided to investigate a possibility to introduce an isobutene pathway deriving from the central metabolite pyruvate by exploiting the L-leucine biosynthesis pathway requiring less acetyl-CoA. In the L-leucine pathway, α-ketoisocaproate (KIC, 2-oxo-4-methylpentanoate) is formed as an intermediate in *Synechocystis*.

The mammalian enzyme α-ketoisocaproate dioxygenase (KICD, EC: 1.13.11.27) catalyzes the decarboxylation and oxygenation of KIC to form HMB in the cytosol of liver cells as a part of the L-leucine catabolic pathway (Sabourin and Bieber, 1982, 1983). The same enzyme, also known as 4-hydroxyphenylpyruvate dioxygenase (HPPD), is responsible for the conversion of 4-hydroxyphenylpyruvate (HPP) to homogentisate (Baldwin et al., 1995). HPPDs are non-heme Fe^2+^-dependent enzymes involved in the degradation pathway of L-tyrosine in a wide range of aerobic organisms from bacteria and plants to animals (Gunsior et al., 2004). The dual physiological function of KICD/HPPD in catabolism of L-leucine and L-tyrosine has been shown in rat and human liver cells (Sabourin and Bieber, 1982; Van Koevering et al., 1992). In many cyanobacteria, such as *Synechocystis*, HPPD is involved in biosynthesis of tocopherols, and the aromatic precursor homogentisate may be used also for the synthesis of plastoquinone (Nowicka and Kruk, 2016).

In the present study, we expressed *Rn*KICD from *Rattus norvegicus* and *Pt*M3K from *Picrophilus torridus* in *Escherichia coli* and investigated the biochemical properties of the purified *Rn*KICD and *Pt*M3K for isobutene formation. We detected isobutene formation both from the purified *Rn*KICD and *Pt*M3K using KIC and HMB as substrates, respectively. We further show the direct conversion of CO_2_ to isobutene *in vivo* by expressing the *Rn*KICD in the cyanobacterium *Synechocystis*.

## 2 Material and methods

### 2.1 Construction of plasmids

The genes encoding α-ketoisocaproate dioxygenase from *Rattus norvegicus* (*Rn*KICD, NCBI Reference Sequence: NP_058929.1) and mevalonate-3-kinase from *Picrophilus torridus* (*Pt*M3K, GenBank accession no. AAT43941.1) were codon optimized for *Synechocystis* and synthesized by GenScript (Table 1, Table S1). The modified pET-N-strep plasmid with a cloning site allowing a fusion to a Strep-tag II on the N-terminus based on pET28a (Novagen) plasmid was used as expression vector for *E. coli*. The codon optimized *Rn*KICD and *Pt*M3K genes were delivered in pUC57 plasmid and digested with *Bgl*II and *Pst*I. The pET-N-strep plasmid was digested with *Bam*HI and *Pst*I fast restriction enzymes. All digested products were purified (DNA Clean & Concentrator-5, Zymo Research) and ligated using the Quick Ligation Kit (New England Biolabs).

**Table 1:** Genetic constructs for heterologous expression and strains used in this study.

Strain	Construct	Description/Expressed gene	Reference
*Synechocystis* sp. PCC 6803			
Syn-EVC	pEEK2-Km^R^	RSF1010-based expression vector with P*trc*-core promoter, BCD2, N-terminal Strep-tag II, Km^R^	Miao et al. (2017)
Syn-*Rn*KICD	pEEK2-*Rn*KICD	*kicD*	This study
Syn-*Pt*M3K	pEEK2-*Pt*M3K	*m3k*	This study
Syn-*Pt*M3K-*Rn*KICD	pEEK2-*Pt*M3K-*Rn*KICD	*m3k, kicD*	This study
Syn-*Pt*M3K-*Rn*KICD-F	pEEK2-*Pt*M3K-*Rn*KICD-F	*m3k-kicD*	This study
			
*E. coli* BL21(DE3)			
pET-N-strep	pET-N-strep	Based on pET28a (Novagen) with modified multiple cloning site and N-terminal Strep-tag II, Km^R^	Englund et al. (2018)
pET-*Rn*KICD	pET-*Rn*KICD	*kicD*	This study
pET-*Rn*M3K	pET-*Rn*M3K	*m3k*	This study

**Fig. 1:**
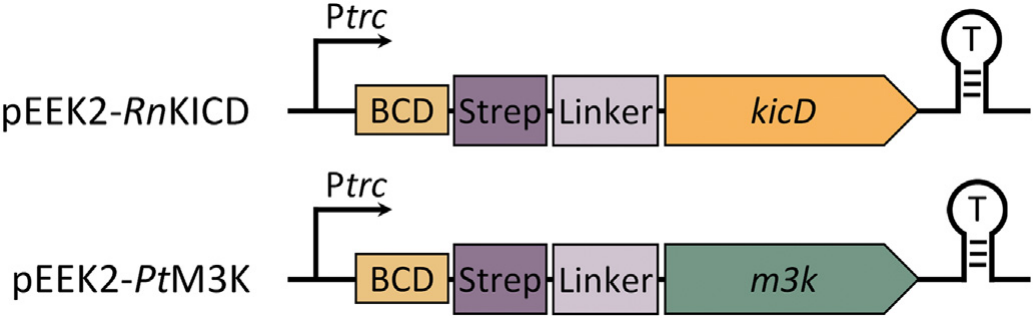
Constructs for expression of *kicD* from *Rattus norvegicus* and *m3k* from *Picrophilus torridus* on the self-replicating vector PEEK2 in *Synechocystis*. The genes are driven by the P*trc*-core promoter with a bicistronic design (BCD). Both proteins were Strep-tagged at the N-terminus.

The pEEK2 (AddGene number: 83492), a broad host range self-replicating vector was used for expression in *Synechocystis* (Miao et al., 2017) (Fig. 1, Table 1). The genes expressed with pEEK2 are under the strong constitutive promoter P*trc*_core_, and the translation initiation is improved with the bicistronic design (BCD) with two Shine-Dalgarno motifs reported by Mutalik et al. (2013). The pEEK2 was digested with *BamH*I and *Spe*I fast restriction enzymes, and the synthetic genes were digested with *Bgl*II and *Spe*I, and ligated together. To construct the operon structure in pEEK2-*Pt*M3K-*Rn*KICD, *Rn*KICD was amplified with primers adding RBS∗, Strep-tag II and a flexible linker and ligated to pEEK-*Pt*M3K plasmid using *Spe*I and *Sac*II sites. To prepare *Pt*M3K-*Rn*KICD fusion protein, pEEK2-*Pt*M3K-*Rn*KICD plasmid was amplified with Phusion Polymerase (Thermo Fisher Scientific) using phosphorylated primers so that only the flexible linker remained between *Rn*KICD and *Pt*M3K sequences. The truncated plasmid with 5′ phosphates on each strand (the PCR product) was treated with *Dpn*I to cleave methylated DNA, and then re-ligated back to circular form resulting in pEEK2-*Pt*M3K-*Rn*KICD-F (Fig. S2A). Primers used in cloning are listed in Table S2.

### 2.2 Strains and cultivation conditions

*Escherichia coli* strain DH5αZ1 was used for subcloning of the constructs and cells were grown in lysogeny broth (LB) medium supplemented with 50 ​μg ​ml^−1^ kanamycin (Km). The plasmids were sequenced (Eurofins) to confirm correct DNA sequence, and the pET-N-Strep based vectors were transformed to *E. coli* BL21 (DE3) and pEEK2 based vectors were conjugated to *Synechocystis*. *E. coli* strain HB101 carrying a conjugal plasmid pRL443 was used for triparental mating (Elhai et al., 1997). The control strain used in this study carries the corresponding empty vector.

*Synechocystis* sp. PCC 6803, a non-motile, glucose-tolerant strain was used as a host for all conjugations. After conjugation and for maintenance, *Synechocystis* was cultivated on agar plates containing BG-11 medium (Rippka et al., 1979). For standard cultivation, BG-11 medium was buffered with 20 ​mM Hepes (pH 7.5) and supplemented with 50 ​μg ​ml^−1^ Km. When indicated in the text BG-11 medium was supplemented with 50 ​mM Hepes-NaOH buffer (pH 7.5) and 50 ​mM NaHCO_3_. Pre-cultures were grown in BG-11 liquid medium in 6-well polystyrene plates. For standard experimental setup, the cultures were adjusted to initial OD_750_=0.1 and grown in 30 ​ml BG-11 in 100 ​ml Erlenmeyer flasks under 30 ​μmol photons m^−2^ ​s^−1^ ​at 30 ​°C and 120 ​rpm shaking. All strains were stored at -80 ​°C, in 10% DMSO. The experiments were started from cryopreserved cells. For high density cultivation (HDC), the cells were grown with HDC 6.10 starter kit (CellDEG GmbH, Berlin). The cultures were adjusted to initial OD_750_=0.3 and 10 ​ml of cell culture was grown in 25 ​ml cultivator vessels in a complete nutrient-rich CD medium according to protocol and conditions described in Dienst et al. (2020), with the exception that no dodecane overlay was used in the vials. The light intensities were increased in sequence: 250 ​μmol photons m^−2^ ​s^−1^ (0h–24h), 490 ​μmol photons m^−2^ ​s^−1^ (24h–48h), 750 ​μmol photons m^−2^ ​s^−1^ (48h–88h). A detailed protocol for HDC for *Synechocystis* can be accessed on protocols.io (https://doi.org/10.17504/protocols.io.9cgh2tw).

### 2.3 Protein expression and purification

Overnight precultures (10 ​ml) of *E. coli* BL21 (DE3) carrying pET-*Rn*KICD or pET-*Pt*M3K were inoculated in 1 ​L of LB media supplemented with 5% of glucose and Km (50 ​μg ​ml^−1^) and grown at 37 ​°C and 150 ​rpm. At an optical density at 600 ​nm of approximately 0.6, protein expression was induced by adding 1 ​mM isopropyl-β-D-1-thiogalactopyranoside (IPTG). Thereafter, cells were grown at 16 ​°C for 16 ​h, collected by centrifugation at 2000 x g for 10 ​min ​at 4 ​°C and resuspended in TBS buffer (100 ​mM Tris-HCl and 150 ​mM NaCl, pH 8.0) containing 25 ​mM MgCl_2_, 10 ​μg ​ml^-1^ of RNase, 10 ​μg ​ml^-1^ of DNase, 60 ​mg ​ml^-1^ lysozyme and Protease Arrest™ Protease Inhibitor Cocktail (100x, G-Biosciences). The cells were subsequently broken by using freezing and thawing cycles. The cell lysate was centrifuged at 55000 ​rpm for 1 ​h, at 4 ​°C, and supernatant was frozen using liquid nitrogen and stored at -80 ​°C.

Before purification, the thawed supernatants were centrifuged at 15000 ​rpm for 15 ​min ​at 4 ​°C. The *Rn*KICD and *Pt*M3K proteins were purified at 4 ​°C using an ÄKTA protein purification system (Cytiva). Crude extracts were applied to a Strep Tag HP column (Cytiva) equilibrated with an equilibration buffer; TBS and 25 ​mM MgCl_2_ pH 8.0. The Strep-tagged proteins were eluted from the column using the equilibration buffer with addition of desthiobiotin to a final concentration of 2.5 ​mM. Elution was monitored by UV (280 ​nm) and 2 ​ml fractions were collected. The protein fractions were concentrated using the Amicon Ultra-15 Centrifugal Filter Units according to manufacturer’s instructions. The concentrated proteins were aliquoted, frozen using liquid nitrogen and stored at -80 ​°C. Fresh aliquots of proteins were used for each experiment.

### 2.4 Protein extraction from *synechocystis* and protein detection

To extract proteins from *Synechocystis*, 2 ​ml of culture was centrifuged at 5000 x g for 10 ​min ​at 4 ​°C. The pellet was washed once and then resuspended with 200 ​μl of buffer containing 50 ​mM Hepes-NaOH, pH 7.5, 30 ​mM CaCl_2_, 800 ​mM sorbitol and Protease Arrest™ Protease Inhibitor Cocktail (100x, G-Biosciences). The cells were disrupted by acid-washed glass beads (425–600 ​μm diameter, Sigma-Aldrich) using the Precellys-24 Beadbeater (Bertin Instruments), program 3 ​× ​30 ​s. Protein concentration was determined with the DC protein assay (Bio-Rad). 10 ​μg of total protein was applied to SDS-PAGE, if nothing else is stated.

Proteins in crude extracts were separated according to their molecular size with Mini Protean TGX Stain free gels (any kDa, Bio-Rad). The gels were run using a Mini-PROTEAN TGX™ system (Bio-Rad) for 1.5 ​h ​at 150 ​V. For visualizing all proteins and to control equal loading, the gels were stained using Page Blue Protein stain (Thermo Scientific). For detecting Strep-tagged proteins, the SDS-gels were blotted to PVDF membranes using the Trans-Blot turbo transfer pack and system (Bio-Rad) according to manufacturer’s instructions. Membranes were blocked in 5% milk for 2 ​h. Detection of the proteins was done using an anti-Strep-tag II antibody (Abcam, ab76949).

### 2.5 Isobutene formation rate from purified enzymes and via spontaneous degradation

#### 2.5.1 Isobutene formation by RnKICD

The reaction mixture (200 ​μl) contained 10 ​μg of purified *Rn*KICD, 2 ​mM of KIC, 100 ​mM Hepes-NaOH buffer (pH 6.0), 1 ​mM FeSO_4_ mixed with 1 ​mM dithiothreitol (DTT) and 0.5 ​mM sodium ascorbate. As a negative control, samples without the addition of enzyme were also analyzed. Reaction mixtures under different conditions were analyzed: (i) pH 7.5, (ii) omitted FeSO_4_ (iii) omitted FeSO_4_, DTT and sodium ascorbate, (iv) added mesotrione (2 ​μM) and (v) added mesotrione (3 ​μM). Assays were initiated by adding the enzyme to the assay mixtures and incubated in 2 ​ml ​GC vials with Teflon coated septa at 30 ​°C for 40 ​min. Three replicate assays were performed for each condition.

#### 2.5.2 Isobutene formation by PtM3K

The reaction mixture (200 ​μl) contained 10 ​μg of purified *Pt*M3K in 100 ​mM Tris-HCl buffer (pH 7.5), 10 ​mM MgCl_2_, 20 ​mM KCl, 10 ​mM ATP and 20 ​mM or 40 ​mM of the substrate HMB. Assays were initiated by adding enzyme to the assay mixtures and incubated in 2 ​ml ​GC vials with Teflon coated septa at 30 ​°C or 50 ​°C for 40 ​min.

#### 2.5.3 Spontaneous degradation of HMB

To analyze the spontaneous degradation rate of HMB, 20 ​mM HMB was dissolved in 0 ​mM, 10 ​mM and 20 ​mM NaOH (pH 3.0, pH 4.2, pH 7.7) in 2 ​ml ​GC vials. Samples were incubated at 22 ​°C, 30 ​°C and 50 ​°C and the headspace was sampled after 7.5 ​h, 4.5 ​h and 3 ​h, respectively. Reaction volume was varied from 200 to 900 ​μl to get above the detection limit. Three replicate assays were performed for each condition.

### 2.6 Measurement of isobutene production rate from *synechocystis*

To analyze the isobutene production rate from *Synechocystis* culture grown in Erlenmeyer flasks, cells were harvested by centrifugation at 5000 ​rpm for 10 ​min ​at RT, at 3 or 7 days depending on the experiment. Then cells were concentrated to OD_750_ ​~ ​4 by resuspending in BG-11 medium supplemented with 50 ​mM Hepes-NaOH buffer (pH 7.5) and 50 ​mM NaHCO_3_ to provide a carbon source. 4 ​ml of cultures were then transferred to 8.4 ​ml gas-tight vials and closed with Teflon coated septa to prevent any absorption of the hydrocarbons to the septum. The gas-tight vials were shaken at 130 ​rpm on a rotary shaker under 80 ​μmol photons m^−2^ ​s^−1^ light for approximately 21 ​h. Additionally Syn-*Pt*M3K and the Syn-EVC strains were supplemented with 20 ​mM or 40 ​mM HMB before incubation in the gas-tight vials. The isobutene production rate was estimated based on OD_750_.

The cells grown in high density cultivation system (HDC) for 3 days were diluted to OD_750_=15 with fresh BG-11 (50 ​mM Hepes pH 7.5, 50 ​mM NaHCO_3_) and 6 ​ml of culture was incubated in gas tight 31 ​ml vials for approximately 20 ​h under 750 ​μmol photons m^−2^ ​s^−1^ and shaken at 320 ​rpm on a rotary shaker. The gas phase was sampled through the Teflon septum with a gas-tight syringe.

### 2.7 Isobutene detection

For detection and quantification of isobutene, headspace gas was analyzed with Clarus 580 Perkin Elmer gas chromatograph (GC) with a packed column (1.8 ​m ​× ​2 ​mm i.d., Cat No. N9305013-ZW5531, Perkin Elmer) fitted with a flame ionization detector. The oven temperature was 190 ​°C for 2.25 ​min, and the carrier gas was N_2_ at a flow rate of 10 ​ml ​min^−1^. The GC retention-time for the isobutene peak from *Rn*KICD and *Pt*M3K enzyme assay and Syn-*Rn*KICD and Syn-*Pt*M3K cells was compared to an authentic isobutene standard (Air Liquide Gas AB, Sweden). Isobutene standard curve was obtained from a dilution series of the isobutene standard and the peak area was converted to a mass value.

### 2.8 Analysis of pyomelanin

Pyomelanin, the oxidation product of homogentisate, was analyzed from the supernatant of *Synechocystis* cultivated in Erlenmeyer-flasks and in the HDC system. The supernatant was diluted 50 times with ddH_2_O and the absorbance spectrum was compared to the oxidation product of commercial homogentisate standard. For alkalization, 10 ​mM homogentisic acid (Sigma-Aldrich) was incubated in 1 ​M NaOH for 1 ​h and diluted 50 times prior to measurement. The change in absorbance of the sample solutions were observed at 200–800 ​nm in a Varian 50 Bio UV–Visible Spectrophotometer in an Eppendorf UVette with a path length of 10 ​mm. Pyomelanin was also visually detected in colonies of pET-*Rn*KICD and pEEK2-*Rn*KICD *E*. *coli* strains.

## 3 Results

### 3.1 Assessing a putative pathway for isobutene production

**Fig. 2:**
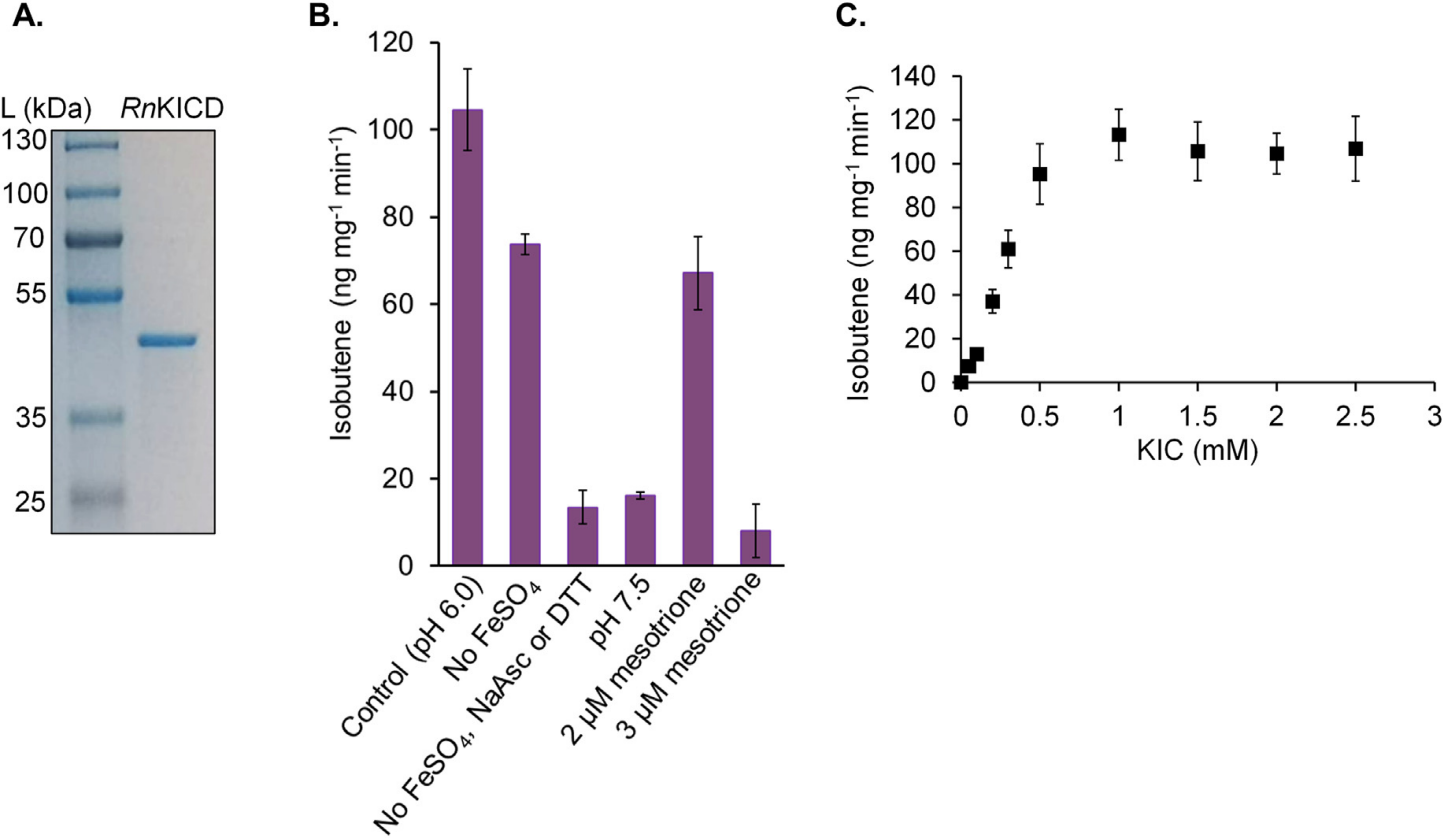
Enzyme characteristics of *Rn*KICD (A) The purified *Rn*KICD from *Rattus norvegicus* subjected to SDS-PAGE and stained with Coomassie blue. (B) Isobutene formation catalyzed by *Rn*KICD. Effect of changing pH, omitting cofactors or adding an inhibitor on the activity of *Rn*KICD. The enzyme assay (Control, pH 6.0) was performed in 100 ​mM Hepes buffer (pH 6.0) containing 1 ​mM FeSO_4_, 1mM DTT and 0.5 ​mM sodium ascorbate (NaAsc). Substrate concentrations of KIC was 2 ​mM and incubation time 40 ​min ​at 30 ​°C. (C) *Rn*KICD activity dependence on substrate concentration. Data shown as mean of three replicate assays; error bars represent standard deviation.

Several engineered pathways were considered for isobutene production. The most prominent pathway to utilize in a photosynthetic oxygenic host seemed to be the L-leucine pathway, since it produces the intermediate metabolite α-ketoisocaproate (KIC). We hypothesized that by expressing an α-ketoisocaproate dioxygenase from *Rattus norvegicus* (*Rn*KICD) and a known isobutene forming enzyme, mevalonate-3-kinase from *Picrophilus torridus* (*Pt*M3K), we would be able to introduce a pathway from the native metabolite KIC to isobutene via HMB. Since *Rn*KICD is oxygen dependent, the bio-catalysis is preferably done in an oxygenic host such as *Synechocystis*. To investigate the potential of *Rn*KICD and *Pt*M3K enzymes for isobutene formation, we first characterized them *in vitro*. The codon optimized genes of *Rn*KICD and *Pt*M3K were cloned into pET-28a based vector and expressed in *E. coli* BL21(D3). Both proteins were fused with N-terminal Strep-tag II and purified from the soluble fraction of the crude extract by affinity chromatography (Fig. 2, Fig. 4A). The purified proteins were tested for their activity for isobutene formation in 2 ​ml ​GC vials with Teflon coated septa. As a short volatile hydrocarbon, isobutene can easily be detected from the headspace by sampling through a gas-tight septum. Enzyme assays were carried out in a 200 ​μl reaction volume containing 10 ​μg of purified protein.

Originally, we intended to combine *Rn*KICD and *Pt*M3K in a coupled enzyme assay for isobutene formation. We, however, discovered that an enzyme assay with only *Rn*KICD formed detectable amount of isobutene in the gaseous headspace. *Rn*KICD catalyzed isobutene formation at a rate of 104.6 ​± ​9 ​ng (mg protein)^-1^ min^-1^ with 2 ​mM KIC as substrate (Fig. 2B). The formation of isobutene by *Rn*KICD was studied by modulating several effectors, including omitting cofactors, changing pH and adding a *Rn*KICD inhibitor. When FeSO_4_ was omitted from the reaction, the isobutene production was reduced by 30%. When FeSO_4_, sodium ascorbate and DTT were omitted from the reaction mixture, isobutene formation diminished by 87%. Changing the pH of the reaction assay from 6.0 to 7.5 reduced the production rate by 84%. Addition of 2 ​μM mesotrione, an inhibitor of *Rn*KICD (Ndikuryayo et al., 2017), reduced the production rate by 36%, and the addition of 3 ​μM mesotrione was enough to almost completely eliminate the *Rn*KICD activity. The addition of 10 ​mM HMB instead of KIC did not yield a detectable amount of isobutene in the headspace (data not shown), indicating that free HMB does not contribute to isobutene formation under this specific assay condition. We further demonstrated the dependence of *Rn*KICD activity on its substrate concentrations (Fig. 2C). Under these conditions *Rn*KICD activity increased with increasing KIC concentration up to approximately 1 ​mM.

**Fig. 3:**
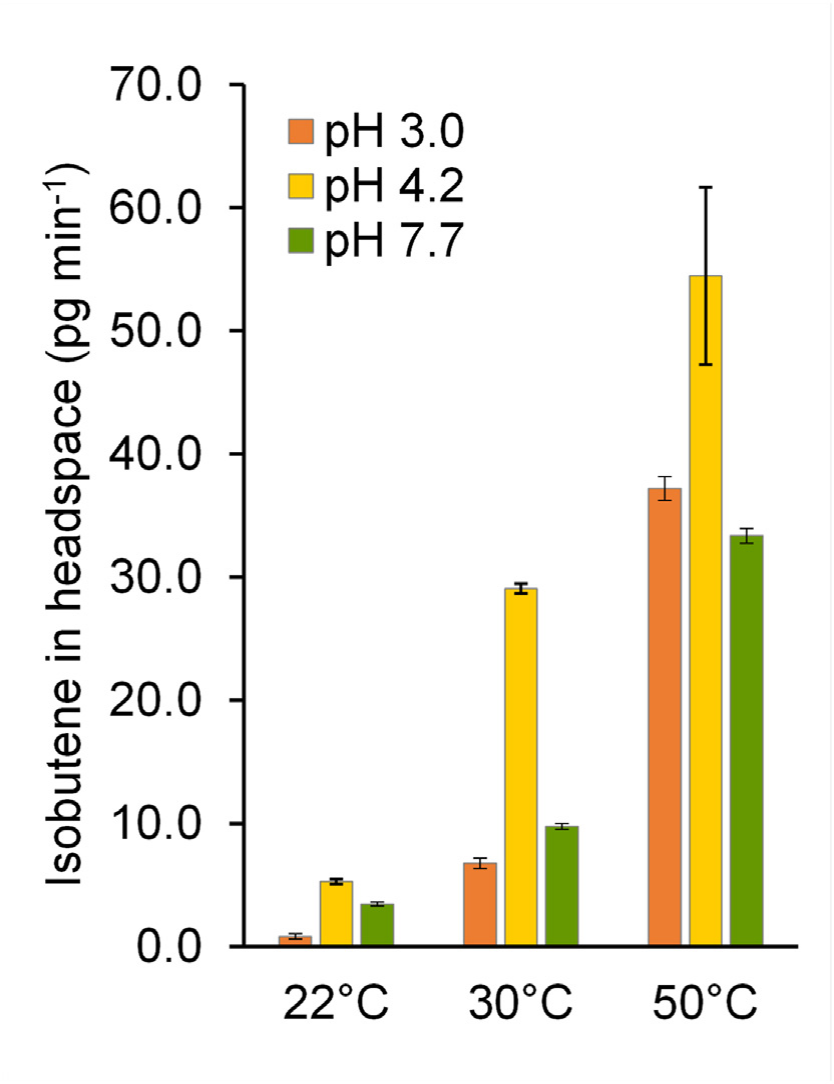
Temperature and pH dependent non-enzymatic isobutene formation from β-hydroxyβ-methylbutyrate (HMB). 20 ​mM HMB was dissolved in 0 ​mM, 10 ​mM and 20 ​mM NaOH (pH 3.0, pH 4.2, pH 7.7) in 2 ​ml ​GC vials. Samples were incubated at 22 ​°C, 30 ​°C and 50 ​°C and the headspace was sampled after 7.5 ​h, 4.5 ​h and 3 ​h, respectively. Data shown as mean of three replicates; error bars represent standard deviation. (For interpretation of the references to color in this figure legend, the reader is referred to the Web version of this article.)

Previously, only HMB has been reported as a product from *Rn*KICD activity on KIC (Sabourin and Bieber, 1982, 1983). It is known that HMB can spontaneously form isobutene via non-enzymatic decomposition (Pressman and Lucas 1940; Rossoni et al., 2015). In order to determine if formation and further decomposition of HMB is the source of isobutene in the enzyme assay performed with *Rn*KICD, we analyzed the isobutene formation rate when HMB was added instead of KIC. For detecting the spontaneous degradation of HMB to isobutene we had to modify the reaction set up, by using a higher concentration of HMB (20 ​mM) and longer incubation times as described in materials and methods 2.5.3. We hypothesized that the degradation rate could be both temperature and pH dependent, and therefore applied three different pH and temperature adjustments. Indeed, the spontaneous formation of isobutene from HMB was temperature and pH dependent (Fig. 3). Highest isobutene accumulation rates, 29.1 ​± ​0.4 ​pg ​min^-1^ and 54.4 ​± ​7 ​pg ​min^-1^, were detected in pH 4.2 ​at 30 ​°C and 50 ​°C, respectively. In comparison, the isobutene accumulation rate in the enzyme assays was 1.0 ​± ​0.1 ​ng ​min^-1^ with 10 ​μg of purified *Rn*KICD and with 2 ​mM KIC as substrate.

**Fig. 4:**
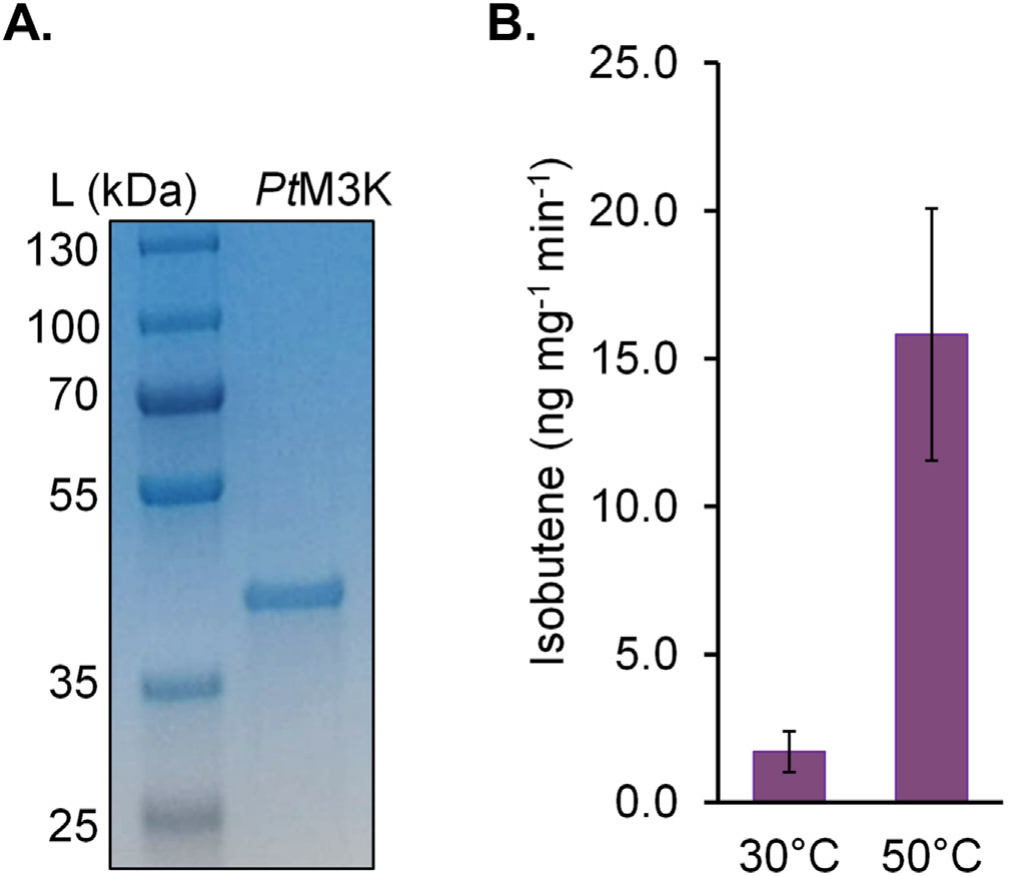
Enzyme characteristics of *Pt*M3K. (A) The purified *Pt*M3K from *Picrophilus torridus* was subjected to SDS-PAGE and detected by Coomassie blue staining. (B) Isobutene formation catalyzed by *Pt*M3K. 40 ​mM HMB was used as substrate and reactions were incubated at pH 7.5, at 30 ​°C or 50 ​°C for 40 ​min. Data shown as mean of three replicate assays; error bars represent standard deviation.

The isobutene formation catalyzed by *Pt*M3K was also analyzed (Fig. 4). The enzyme assay for *Pt*M3K was performed in buffer described in Rossoni et al. (2015). In addition, 10 ​mM ATP was added, since *Pt*M3K is an ATP-dependent enzyme. With 40 ​mM substrate concentrations of HMB, *Pt*M3K was calculated to produce isobutene with the rate of 1.71 ​± ​0.7 ​ng (mg protein)^-1^ ​min^-1^ ​at 30 ​°C and 15.8 ​± ​4.3 ​ng (mg protein)^-1^ ​min^-1^ ​at 50 ​°C (Fig. 4B). The isobutene that was formed non-enzymatically from HMB in control reactions was subtracted from the values. If ATP was omitted from the reactions the isobutene formation rate of the assays including *Pt*M3K was similar to the reaction without the enzyme.

### 3.2 Isobutene production from engineered *synechocystis* strains

**Fig. 5:**
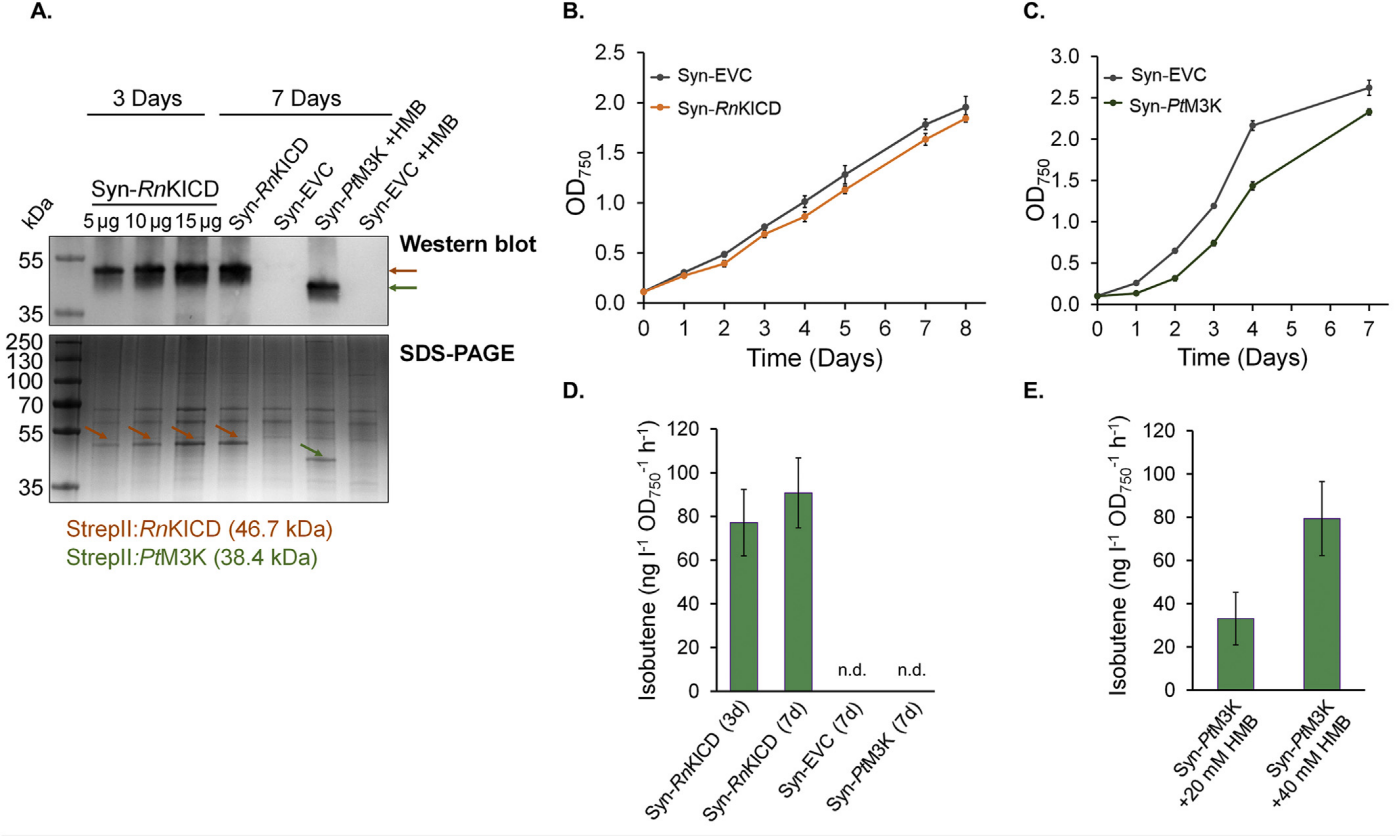
Isobutene production and growth of isobutene producing and control *Synechocystis* strains. (A) SDS-PAGE and Western blot analysis of the recombinant proteins *Rn*KICD and *Pt*M3K extracted from *Synechocystis*. For *Rn*KICD (3d) three different concentrations were loaded on the gel (5 ​μg, 10 ​μg and 15 ​μg). All other samples were loaded with 10 ​μg of total protein. *Rn*KICD and *Pt*M3K proteins are marked with orange and green arrows, respectively. This was repeated for two individual gels. The 2^nd^ gel was used for Western blot analysis with an anti-Strep-tag II antibody. (B) Growth of the Syn-*Rn*KICD and Syn-EVC strains under 30 ​μmol photons m^−2^ ​s^−1^, in BG11 with 20 ​mM Hepes at pH 7.5 (C) Growth of the Syn-*Pt*M3K and Syn-EVC strains under 30 ​μmol photons m^−2^ ​s^−1^, in BG11 with 50 ​mM Hepes at pH 7.5 and 50 ​mM NaHCO_3_. Isobutene production rates for Syn-*Rn*KICD (D) and Syn-*Pt*M3K (E). Syn-*Rn*KICD cells were grown for 3 and 7 days and Syn-*Pt*M3K for 7 days, harvested and resuspended in fresh BG-11 with addition of 50 ​mM Hepes-NaOH pH 7.5 and 50 ​mM NaHCO_3_. 20 ​mM or 40 ​mM HMB was added to Syn-*Pt*M3K and Syn-EVC cells in the assay vials. The isobutene formed by Syn-EVC and accumulated in the headspace of the vials was considered as non-enzymatic, and the value obtained was subtracted from the samples of Syn-*Pt*M3K. Data shown as mean of three biological replicates; error bars represent standard deviation. (For interpretation of the references to color in this figure legend, the reader is referred to the Web version of this article.)

To determine if *Rn*KICD and *Pt*M3K were active *in vivo*, the enzymes were heterologously expressed in *Synechocystis* and the engineered strains were tested for isobutene production. The codon optimized *Rn*KICD and *Pt*M3K genes were expressed under a strong constitutive P*trc*_core_ promoter with a BCD2 construct (Mutalik et al., 2013) in a self-replicating pEEK2-plasmid in WT strain of *Synechocystis* (Fig. 1). Three strains were generated: Syn-*Rn*KICD (*kicD*), Syn-*Pt*M3K (*m3k*) and Syn-EVC (empty vector control). Expression of *Rn*KICD and *Pt*M3K proteins in *Synechocystis* was confirmed with SDS-PAGE electrophoresis and Western blot via antibody detection against a Strep-tag II fused to the N-terminus of the proteins (Fig. 5A).

Syn-*Rn*KICD, Syn-*Pt*M3K and Syn-EVC were cultivated in Erlenmeyer flasks under 30 ​μmol photons m^−2^ ​s^−1^ light. The culture of Syn-*Pt*M3K, which showed slower growth and a bleaching phenotype under standard growth conditions (Fig. S1A-C), was supplemented with 50 ​mM NaHCO_3_ in the Erlenmeyer flasks. The cells were subsequently sampled at linear growth phase (3 days) or at stationary growth phase (7 days) (Fig. 5B and C). Prior analysis, the cultures were concentrated, and resuspended to OD_750_≈ ​4 in BG-11 medium supplemented with 50 ​mM NaHCO_3_ to provide a carbon source in the gas-tight vials and with 50 ​mM Hepes-NaOH pH 7.5 to prevent the increase of the pH in the culture. The vials were shaken under 80 ​μmol photons m^−2^ ​s^−1^ light for approximately 21 ​h. The vials were sealed with Teflon coated septa to prevent any absorption of the hydrocarbons to the septa. The headspace was sampled through septa and analyzed with gas chromatography. In the *Rn*KICD expressing strain, a peak specific to isobutene was detected while no peak was detected in EVC or *Pt*M3K expressing strains. Syn-*Rn*KICD produced 77.2 ​± ​15 ​ng isobutene l^-1^ OD_750_^-1^ h^-1^ and 91 ​± ​16 ​ng ​l^-1^ OD_750_^-1^ h^-1^ after 3 and 7 days of cultivation, respectively (Fig. 5D).

The isobutene production in Syn-*Pt*M3K was tested by supplementing the *Synechocystis* cultures in gas tight vials with exogenous HMB. The isobutene detected from Syn-*Pt*M3K strain was 33.1 ​± ​12 ​ng ​l^-1^ OD_750_^-1^ h^-1^ with 20 ​mM HMB as substrate and 79.4 ​± ​17 ​ng ​l^-1^ OD_750_^-1^ h^-1^ with 40 ​mM HMB as substrate (Fig. 5E). 20 ​mM and 40 ​mM HMB was provided also for Syn-EVC, and the isobutene formed non-enzymatically was analyzed and subtracted from the values obtained for Syn-*Pt*M3K during the same incubation time.

The *in vitro* synthesis of isobutene using the coupled reaction of *Rn*KICD and *Pt*M3K did not form any isobutene under the conditions used, most likely because the amount of HMB produced by *Rn*KICD was too low to support the activity of *Pt*M3K, and the temperature optima of the two enzymes differs. The situation might be different *in vivo* with constant flow through pathway intermediates to isobutene. Therefore, we additionally constructed a Syn-*Pt*M3K-*Rn*KICD strain expressing both *Pt*M3K and *Rn*KICD from a synthetic operon. In addition, to investigate if we could enable an efficient channeling of the putative HMB intermediate, a *Pt*M3K-*Rn*KICD fusion protein was expressed in Syn-*Pt*M3K-*Rn*KICD-F strain (Fig. S2A). The successful expression of *Pt*M3K-*Rn*KICD fusion protein was shown in stained SDS-PAGE, together with some cleavage products (Fig. S2D). The Syn-EVC, Syn-M3K, Syn-*Rn*KICD, Syn-*Pt*M3K-*Rn*KICD and Syn-*Pt*M3K-*Rn*KICD-F were all grown in BG-11 medium supplemented with 50 ​mM Hepes-NaOH pH 7.5 and 50 ​mM NaHCO_3_ (Fig. S2B). Unlike in previous experiments, NaHCO_3_ was provided to all flask cultures to avoid reduced pigmentation as seen for the Syn-*Pt*M3K strain (Fig. S1). The isobutene production rates were measured after 3 and 7 days of growth. Both Syn-*Pt*M3K-*Rn*KICD and Syn-*Pt*M3K-*Rn*KICD-F strains produced approximately four times less isobutene than the *Syn*-*Rn*KICD strain (Fig. S2C). The amount of *Rn*KICD in Syn-*Pt*M3K-*Rn*KICD strain, in which the corresponding gene is expressed from the operon, was not as high as the amount of *Rn*KICD in the Syn-*Rn*KICD strain, as shown by SDS-PAGE (Fig. S2D). This might explain why the production of isobutene from Syn-*Pt*M3K-*Rn*KICD was less than from *Syn*-*Rn*KICD.

### 3.3 Specific and volumetric isobutene productivity in a small-scale high-density cultivation system

In order to investigate if a different cultivation strategy would increase the isobutene formation, the Syn-*Rn*KICD and Syn-EVC strains were grown in a small-scale high density cultivation (HDC) system, as described by Dienst et al. (2020). The authors showed increased volumetric productivity of sesquiterpenes when engineered *Synechocystis* strains were grown in a HDC system that maintains relatively high concentration of CO_2_ due to diffusion through a hydrophobic membrane from a carbonate buffer provided from underneath the culture vials. The Syn-*Rn*KICD cultivated in HDC reached OD_750_=30 on the third day, then cells were diluted to OD_750_=15 with fresh BG-11 medium supplemented with 50 ​mM NaHCO_3_. 6 ​ml culture was incubated in 31 ​ml vials for approximately 20 ​h under 750 ​μmol photons m^−2^ ​s^−1^ ​at 320 ​rpm. Under this specific cultivation set up the HDC volumetric productivity was approximately two times higher as compared to the cells cultivated in Erlenmeyer flasks, 937 and 421 ​ng isobutene l^-1^ h^-1^, respectively (Table 2). However, the specific productivity normalized to cell density was lower, 42 ​ng ​l^−1^ OD_750_^−1^ ​h^−1^ in HDC system compared to 91 ​ng ​l^−1^ OD_750_^−1^ ​h^−1^ in flask cultivation.

**Table 2:** Isobutene volumetric and specific productivity of Syn-*Rn*KICD in Erlenmeyer flasks (7 days) and HDC system (3 days).

Cultivation system	End OD_750_	Vial (ml)	Culture volume (ml)	Incubation time (h)	Light (μmol photons m^−2^ ​s^−1^)	Isobutene (ng l^−1^ ​h^−1^)	Isobutene (ng l^−1^ OD_750_^−1^ ​h^−1^)
**Flask**	5.8 ​± ​0.1	8.4	4	21	80	421 ​± ​76	91 ​± ​16
**HDC**	22.0 ​± ​3.4	31.0	6	20	750	937 ​± ​283	42 ​± ​6.5

### 3.4 RnKICD might function in two pathways in engineered *synechocystis*

It was noted that the supernatant of the HDC of Syn-*Rn*KICD, unlike the supernatant of Syn-EVC, was colored with brown pigment (Fig. S3A). From the supernatant of the standard Erlenmeyer flask cultivation no difference between strains was observed. The culture of *E. coli* strains carrying pEEK-*Rn*KICD plasmid was also turning visibly brown (data not shown). As *Rn*KICD has been shown to have HPPD activity, it is likely that the pigment formation is due to catalytic conversion of HPP to homogentisate by *Rn*KICD/HPPD in Syn-*Rn*KICD. Several studies have reported the brown pigmentation as a characteristic for HPPD enzyme activity (Lee et al., 1996; Keon and Hargreaves 1998). The reaction is due to oxidation and subsequent polymerization of the homogentisate to pyomelanin pigment (Zannoni et al., 1969; Denoya et al., 1994; Schmaler-Ripcke et al., 2009). We compared the absorbance spectrum of the Syn-*Rn*KICD supernatant to an oxidized homogentisate standard. The oxidized homogentisate displayed an increase in absorbance from 550 to 250 ​nm as compared to reduced homogentisate standard (Fig. S3B-C). The absorbance peak at 290 ​nm represents non-oxidized homogentisate (Zhu et al., 2015). An increase in absorbance was also identified when the supernatants of Syn-*Rn*KICD was compared to Syn-EVC (Fig. S3C). The absorbance spectrum of 50 times diluted supernatant of Syn-*Rn*KICD showed higher absorbance at wavelengths below 550 ​nm as compared to Syn-EVC. The increase in absorbance around 400–440 ​nm is characteristic for accumulation of pyomelanin pigmentation resulting from the oxidation of homogentisate (Lee et al., 2008; Schmaler-Ripcke et al., 2009). Although the absorption spectrum does not give a definite identification of the pigment, the pigmentation is recognized in the literature as pyomelanin and a direct product of the activity of HPPD. This is also supported by the spectrum of pyomelanin from the *in vitro* oxidation of HGA (Fig. S3C). In addition to the direct effect of the high cell density also other cultivation conditions, such as higher light intensities applied to the HDC could influence the pigment concentrations in Syn-*Rn*KICD cultures.

## 4 Discussion

**Fig. 6:**
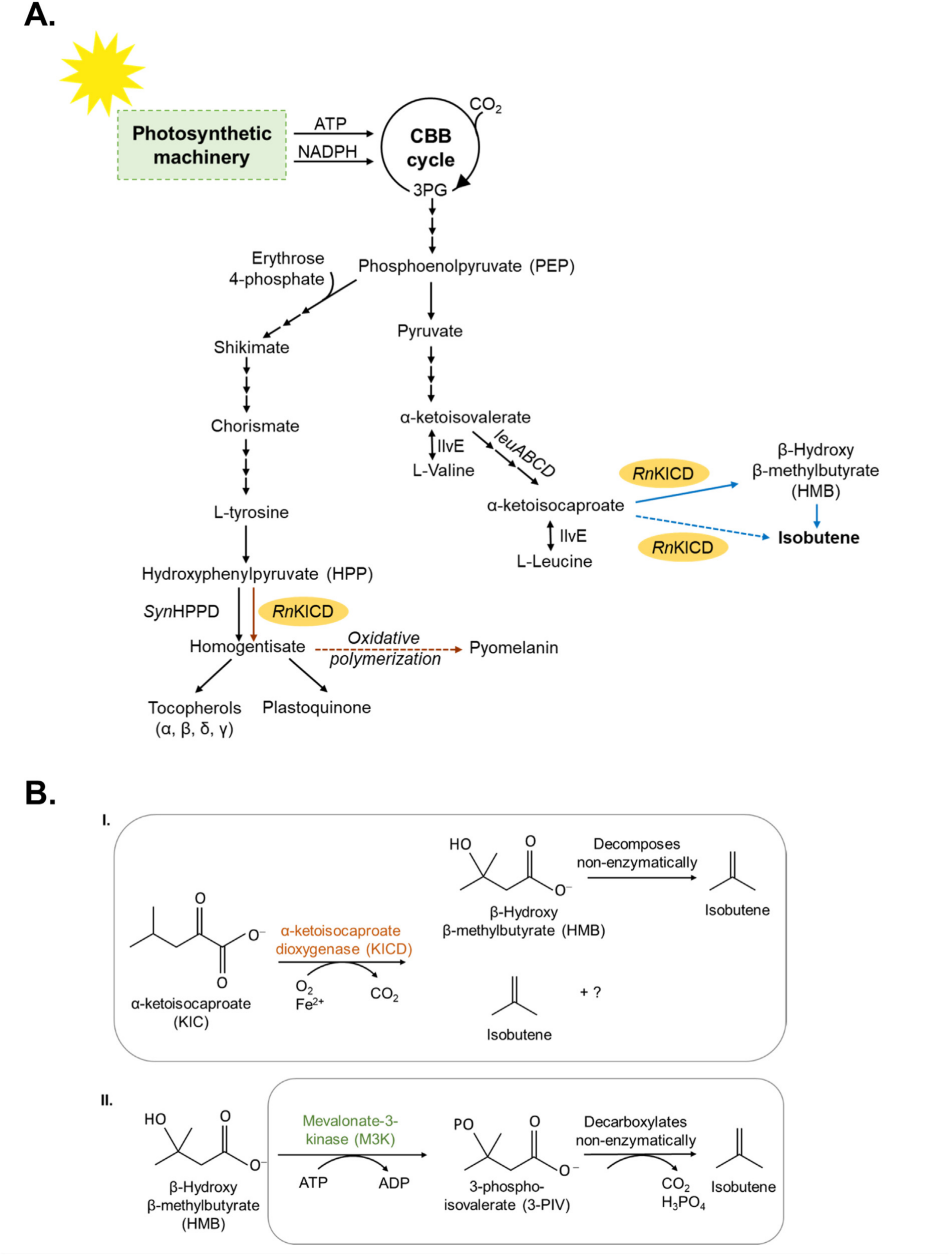
The isobutene biosynthesis pathways in engineered *Synechocystis*. (A) Putative pathway for biosynthesis of β-Hydroxy-β-methylbutyrate (HMB), isobutene and homogentisate (HGA) in *Synechocystis* by introducing α-ketoisocaproate dioxygenase from *Rattus norvegicus* (*Rn*KICD). Black arrows indicate native pathways, blue and brown arrows non-native pathways. Dotted blue arrow indicates the reaction catalyzed by *Rn*KICD suggested in this study and dotted brown arrows indicate a visible formation of pyomelanin in Syn-*Rn*KICD. (B) Production of isobutene via two introduced enzymes: (I.) *Rn*KICD or (II.) Mevalonate-3-Kinase (*Pt*M3K) from *Picrophilus torridus*. Boxes depict the *in vivo* enzyme activities shown in this study. (For interpretation of the references to color in this figure legend, the reader is referred to the Web version of this article.)

In this work we demonstrate that by introducing a synthetic pathway consisting of one heterologously-expressed enzyme, *Rn*KICD, the cyanobacterium *Synechocystis* re-routes the central metabolism from pyruvate via the L-leucine biosynthesis pathway-derived metabolite α-ketoisocaproate (KIC) to isobutene (Fig. 6). Cyanobacteria have shown to be a promising platform for direct phototrophic conversion of CO_2_ to different biofuels such as alcohols, hydrocarbons and hydrogen. One limiting factor for microbial biofuel production is product toxicity. At higher concentration, hydrocarbons may alter membrane fluidity and permeability, and thus compromise the membrane integrity (Ruffing and Trahan 2014; Sikkema et al., 1995). Producing short volatile hydrocarbons that quickly escape the cells and growth medium should minimize product toxicity. Volatile compounds can also be collected from the headspace and thus avoid the costly extraction of the product from the culture. To date, only a few volatile compounds have been produced in cyanobacteria, such as hydrogen, ethylene and isoprene (for review, see Knoot et al., 2018). To our knowledge, this is the first study aiming to identify possible pathways and to build a cyanobacterial chassis for isobutene production.

Expression platforms for synthetic pathways in heterotrophic bacteria cannot always be directly implemented in photoautotrophic cyanobacteria. In *E. coli*, as a facultative anaerobe, fermentative pathways under oxygen-limited conditions enable efficient conversion of sugars to reduced metabolites valuable for biofuels such as ethanol, isobutanol and butanol (Trinh et al., 2011; van Leeuwen et al., 2012). Likewise, a preferred pathway leading to isobutene formation in *E. coli* derives from fermentative pathway via acetone and acetyl-CoA (Marlière 2011b). In cyanobacteria, however, the cellular content of pyruvate is significantly higher than acetyl-CoA under light (Dempo et al., 2014). Therefore, our strategy was to produce isobutene using a pathway less dependent on acetyl-CoA. We targeted the isobutene pathway to share a precursor KIC from the amino acid L-leucine biosynthesis pathway deriving from pyruvate in *Synechocystis*. The intracellular concentration of KIC was recognized to be high enough to sustain isobutene production in Syn-*Rn*KICD strain.

In an attempt to improve the isobutene production, we designed a synthetic pathway consisting of *Rn*KICD and *Pt*M3K in *Synechocystis*. The rationale behind this is that earlier studies have shown that *Rn*KICD catalyzes the formation of HMB from KIC (Sabourin and Bieber, 1981), and *Pt*M3K catalyzes the formation of isobutene from HMB (Rossoni et al., 2015). One strategy was to express the enzyme from a two gene operon resulting in the strain Syn-*Pt*M3K-*Rn*KICD, and in a second strategy a fusion protein of the two enzymes was constructed, resulting in the Syn-*Pt*M3K-*Rn*KICD-F strain. We discovered that neither of these strains produced isobutene at a higher rate than Syn-*Rn*KICD (Fig. S2C), indicating that these two enzymatic reactions are not directly compatible for an efficient pathway from KIC to isobutene via the putative intermediate HMB in *Synechocystis*. The reasons for this could at least in part be explained by the results from the *in vivo* analysis of Syn-*Pt*M3K and Syn-*Rn*KICD. Syn-*Rn*KICD reached a productivity of 91 ​ng ​l^-1^ OD_750_^-1^ h^-1^. In contrast, Syn-*Pt*M3K required 40 ​mM externally added HMB to reach an isobutene production rate similar in magnitude. The extent of HMB taken in by the cells could not be analyzed, but 20 ​mM HMB was required to determine whether *Pt*M3K is active *in vivo*, indicating that *Pt*M3K is one bottleneck for the coupled isobutene producing pathway. This was also supported by results from the *in vitro* analyses. The isobutene formation rate of *Rn*KICD was determined to be 104.6 ​± ​9 ​ng (mg protein)^-1^ min^-1^ with 2 ​mM KIC (Fig. 2B), while for *Pt*M3K catalysis 40 ​mM of the substrate HMB was needed to reach an isobutene formation rate of 1.7 ​± ​0.7 ​ng (mg protein)^-1^ ​min^-1^ ​at 30 ​°C (Fig. 4B). This result is in line with previously reported by Rossoni et al. (2015) (Table 3). Taken together, we conclude that it is likely that the activity of *Rn*KICD does not generate enough HMB to serve as substrate for *Pt*M3K, and thus most – if not all – isobutene formed derives from *Rn*KICD activity. Another reason for the low activity is that the temperature optimum of *Pt*M3K is not supported by the *in vivo* conditions of *Synechocystis*. From our work, we cannot conclude about the *in vivo* function of *Pt*M3K in *Synechocystis*, but we observed a stressed phenotype in the strain expressing *Pt*M3K (Fig. S1). It is likely that the *Pt*M3K catalyzes phosphorylation of an unidentified substrate other than HMB in *Synechocystis*. None of the substrates for *Pt*M3K reported by Rossoni et al. (2015) are native for *Synechocystis* and the connection to the phenotype is not known. This observation serves as an example of unexpected effects when a heterologously expressed enzyme behaves differently in *E. coli* compared to in a photoautotrophic host.

**Table 3:** Specific activities, substrates and cofactors of isobutene-forming enzymes and isobutene production rates from microbial hosts. *Sc*MDD is from *Saccharomyces cerevisiae*, *Pt*M3K from *Picrophilus torridus* and *Cm*P450 from *Cystobasidium minutum.* Wcw, wet cell weight; dcw, dry cell weight.

Enzyme	Name	Purified enzyme			Whole cells		
		Substrate	Cofactors	Isobutene formation rate (ng mg^-1^ min^-1^)	Isobutene production rate	Production host	Reference
Diphosphomevalonate decarboxylase (R74H)	*Sc*MDD2 (R74H)	333 ​mM HMB	250 ​mM ATP	0.36	0.33 ​μg ​g cells^-1^ h^-1^ wcw	*E. coli*	Gogerty and Bobik (2010)
Diphosphomevalonate decarboxylase	*Sc*MDD (WT)	50 mM HMB	40 ​mM ATP	0.13	0.025 ​μg ​g cells^-1^ h^-1^ wcw	*E. coli*	Rossoni et al. (2015)
Mevalonate-3-kinase	*Pt*M3K	50 ​mM HMB	40 ​mM ATP	1.5^a^/162^b^	1.70 ​μg ​g cells^-1^ h^-1^ wcw	*E. coli*	Rossoni et al. (2015)
Cytochrome P450	*Cm*P450	66 ​mM isovalerate	400 ​mM NADPH, O_2_	269	41 ​μg ​g cells^-1^ h^-1^ dcw	*C. minutum*	Fujii et al. (1987), Fukuda et al., 994
Mevalonate-3-kinase	*Pt*M3K	40 ​mM HMB	10 ​mM ATP	1.71 ​± ​0.7^a^/15.8 ​± ​4.3^b^	79 ​ng ​l^−1^ OD_750_^−1^ ​h^−1^	*Synechocystis*	This study (7d in a Flask)
α-ketoisocaproate dioxygenase	*Rn*KICD	2 ​mM KIC		104.6 ​± ​9	91 ​ng ​l^−1^ OD_750_^−1^ ​h^−1^ (without added exogenous substrate)	*Synechocystis*	This study (7d in a Flask)

a The enzyme assay was incubated at 30 ​°C.

b The enzyme assay was incubated at 50 ​°C.

Although *Rn*KICD has been studied earlier, then as an HMB-forming enzyme, the catalytic mechanisms of *Rn*KICD in isobutene formation is still to be resolved. Sabourin and Bieber (1981) identified the major product of the reaction catalyzed by *Rn*KICD to be HMB. This was shown by using isotope labelled KIC as a substrate and by investigating the fate of ^3^H and ^14^C labelled products, not taking volatile products into account. According to the estimation, up to 80% of the substrate was converted to HMB in their reaction conditions. Since HMB can decompose spontaneously to isobutene (Fig. 3) a reasonable route for isobutene formation would be starting from KIC via KICD-catalysis to HMB followed by a spontaneous decay to isobutene. However, a spontaneous formation can explain only a fraction of the isobutene formed by *Rn*KICD, since the addition of 10 ​mM HMB to the *Rn*KICD enzyme assay without added KIC as substrate did not form any detectable amount of isobutene.

The mechanism of *Rn*KICD in isobutene formation could not be determined by comparisons to earlier described isobutene-forming enzymes such as *Pt*M3K, *Sc*MDD and cytochrome P450s. These enzymes differ from *Rn*KICD in co-factor dependence (Table 3), and there are no conserved protein domains that indicates evolutionary relations. We suggest that the *Rn*KICD is substrate and product promiscuous, and both HMB and isobutene are formed from the same substrate, KIC (Fig. 6B). The reaction could be concerted or comprise of two subsequent reactions (stepwise). One example of product promiscuity in an oxygenase enzyme was reported for naphthalene 1,2-dioxygenase, which has been shown to form three products from the same substrate ethylbenzene in varying ratios (Ferraro et al., 2017). In addition, the spontaneous decay of HMB was shown to be pH dependent, and faster in pH 4.2 compared to more acidic or more alkaline pH (Fig. 3). This information could potentially be useful and provide insight of a possible mechanism of catalysis of KIC to isobutene via HMB within the active pocket of *Rn*KICD.

For larger scale applications, further metabolic engineering and genetically-stable production strains, as well as development of more efficient cultivation and gas capturing systems are required (Moulin et al., 2019).

Several enzymes utilized in metabolic engineering for production are promiscuous for substrate specificity, which is a challenge when designing heterologous pathways. Previous reports have shown dual physiological function for *Rn*KICD with catalytic activity both on KIC and HPP as substrates (Sabourin and Bieber, 1982). In the cytosol of rat liver and kidney, *Rn*KICD converts HPP to homogentisate as part of L-tyrosine catabolism, and in addition it functions in detoxifying KIC by converting it to the non-toxic HMB, which is further excreted in urine (Sabourin and Bieber, 1982; Van Koevering et al., 1992). Likewise, *Rn*KICD in *Synechocystis* is likely involved in both the synthetic isobutene pathway and endogenous shikimate pathway, and thus production titers might be affected (Fig. 6A and S3). One approach to increase substrate specificity of *Rn*KICD is by using rational protein engineering which has been demonstrated to be valuable to increase production titer. One recent example is improved isobutanol synthesis in *Synechocystis* by modifying the α-ketoisovalerate decarboxylase (Miao et al., 2018).

Another challenge that should be overcome is the genetic instability, as an effect of heterologous expression of enzymes in cyanobacteria (Pérez et al., 2019; Jones, 2014). This might cause a reduction in the biosynthesis of the product of interest, which was observed for Syn-*Rn*KICD during prolonged growth over weeks as reduced isobutene production (data not shown). Although well-known, genetic instability in cyanobacteria is sparsely studied, and the suggested reasons for it are diverse (Jones, 2014). In our expression system, the strong constitutive promoter or the autonomously replicating plasmid construct might be the reasons. In future applications, genomic integration or driving the expression of *Rn*KICD and/or *Pt*M3K with an inducible promoter could be beneficial to minimize the negative implications of isobutene production to growth of *Synechocystis*.

In addition, a platform for cultivating cyanobacteria at a high-density is of a significant interest, and has been developed in recent years for achieving higher production titers and rates (Dienst et al., 2020). Indeed, the highest volumetric production rate of isobutene by Syn-*Rn*KICD was reached with a HDC system, yielding 937 ​ng ​l^−1^ ​h^−1^ (Table 2). Dehm et al. (2019) suggested that HDC has a positive effect of the biosynthesis of various metabolites in cyanobacteria due to the high intrinsic CO_2_ level, the high light and the decreased feedback of the wanted metabolites due to fast growth. The main benefit of using the small scale HDC system in a research lab is the reduced cultivation time needed for screening of cyanobacterial strains producing isobutene and other valuable chemicals.

In conclusion, we identified a novel biocatalyst, *Rn*KICD, for isobutene formation from CO_2_. The heterologous expression of *Rn*KICD in *Synechocystis* is all that is needed to enable isobutene production. By introduction of *Rn*KICD the carbon flow from pyruvate was redirected to isobutene via an intermediate metabolite, KIC in the L-leucin pathway. Further optimization of the pathway is needed to improve the isobutene productivity and titer. Our finding demonstrates the importance of identifying novel enzyme candidates for bacterial production of volatile hydrocarbons. This study serves as a proof of concept using photosynthetic microorganism for manufacturing of isobutene.

## Funding

This work was supported by 10.13039/501100004527Swedish Energy Agency (project no. 44728-1); and the NordForsk Nordic Center of Excellence ‘NordAqua’ (project no. 82845).

## Credit author statement

**Henna Mustila**: Methodology, Validation, Investigation, Vizualisation, Writing-Original Draft. **Amit Kugler**: Writing-Review & Editing, Validation. **Karin Stensjö**: Conceptualization, Supervision, Project administration, Writing-Review & Editing

## Declaration of competing interest

The authors declare that they have no competing interests.
