# Supplementary material for: Isobutene production in Synechocystis sp. PCC 6803 by introducing α-ketoisocaproate dioxygenase from Rattus norvegicus
Source: Metab Eng Commun. 2021 Jan 23;12:e00163. doi: 10.1016/j.mec.2021.e00163 (PMC7856465; doi:10.1016/j.mec.2021.e00163)
Supplement: Multimedia component 1 [file mmc1.docx]

**Supplementary Information for**

Isobutene production in *Synechocystis* sp. PCC 6803 by introducing α-ketoisocaproate dioxygenase from *Rattus norvegicus*

Henna Mustila^a,1^, Amit Kugler^a^ Karin Stensjö^a^*

^a^Microbial chemistry, Department of Chemistry-Ångström Laboratory, Uppsala University, SE-751 20 Uppsala, Sweden

^1^Present address: Molecular Plant Biology, Department of Life Technologies, University of Turku, Turku FI-20014, Finland

^*^Corresponding author.

*E-mail addresses*: karin.stensjo@kemi.uu.se (K. Stensjö).

**Fig. S1.** Pigments in engineered *Synechocystis* strains.

**
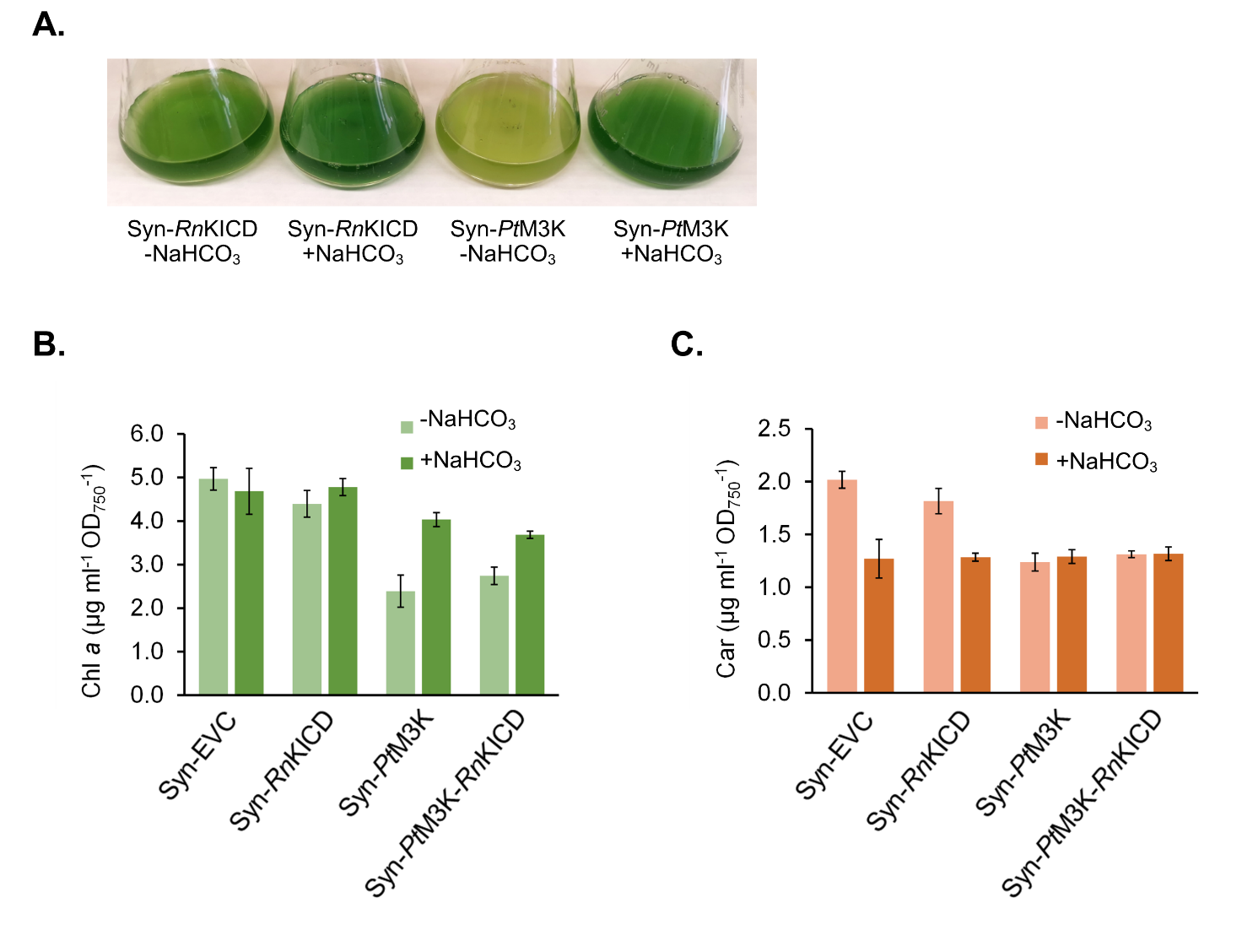
**

**Fig. S1.** Pigments in engineered *Synechocystis* strains. (A) Syn-*Rn*KICD and Syn-*Pt*M3K strains grown under 30 µmol photons m^−2^ s^−1^, BG11 with 50 mM Hepes pH 7.5 and 50 mM NaHCO_3_ (+) or with 20 mM Hepes pH 7.5, but without NaHCO_3_ (­–). (B) Chlorophyll *a* and (C) carotenoids analysed from methanol extracts of engineered *Synechocystis* strains. Chlorophyll *a* and carotenoids content was analysed according to Zavřel et al. (2015). Cells were grown for 4 days prior to extraction. Data shown as mean of three biological replicates; error bars represent standard deviation.

**Fig. S2**. *Synechocystis* strains engineered for isobutene production.


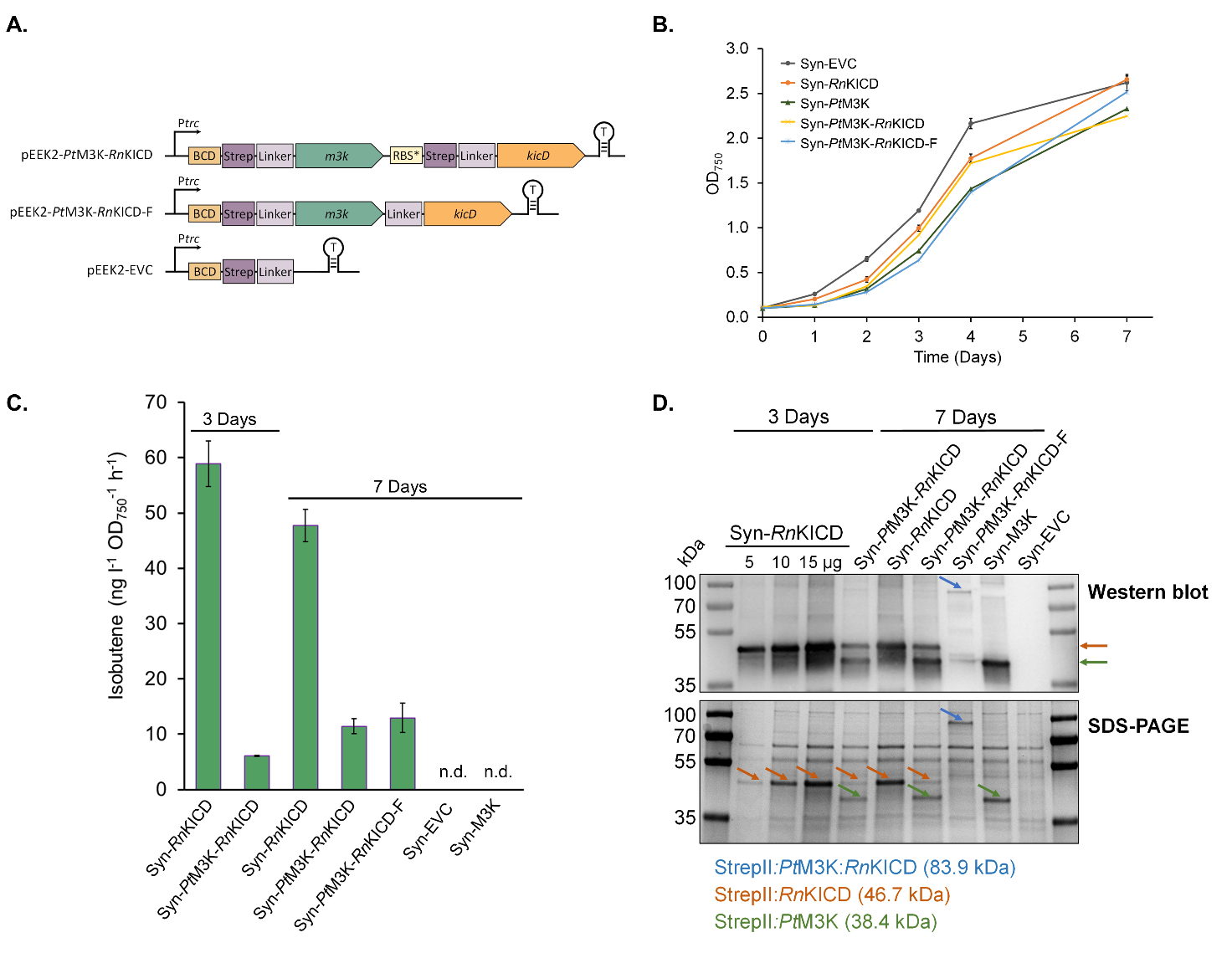


**Fig. S2**. *Synechocystis* strains engineered for isobutene production. (A) Constructs for Syn-M3K-KICD strain expressing both *Pt*M3K and *Rn*KICD or Syn-*Pt*M3K-*Rn*KICD-F strain expressing *Pt*M3K-*Rn*KICD fusion protein. Expression on a self-replicating vector in *Synechocystis*. (B) Growth and (C) isobutene production rates of the engineered *Synechocystis* strains. Cells were grown for 3 and 7 days in BG-11 with addition of 50 mM Hepes-NaOH pH 7.5 and 50 mM NaHCO_3_. These cultivation conditions differ from the ones used for the results presented in Fig 2. (D) SDS-PAGE and Western blot analysis of the recombinant proteins *Rn*KICD, *Pt*M3K and *Pt*M3K-*Rn*KICD fusion protein extracted from *Synechocystis*. For *Rn*KICD (3d) three different concentrations were loaded on the gel (5 µg, 10 µg and 15 µg). All other samples were loaded with 10 µg of total protein. *Rn*KICD and *Pt*M3K proteins are marked with orange and green arrows, respectively and *Pt*M3K-*Rn*KICD fusion protein is indicated by a blue arrow. This was repeated for two individual gels. The 2^nd^ gel was used for Western blot analysis with an anti-Strep-tag II antibody.

**Reference**

Zavřel, T., Sinetova, M. A. and Červený, J., 2015. Measurement of chlorophyll a and carotenoids concentration in cyanobacteria. Bio-protocol 5:e1467. DOI: 10.21769/BioProtoc.1467.

**Fig. S3.** Pyomelanin formation in high density cultivation (HDC) system.

**
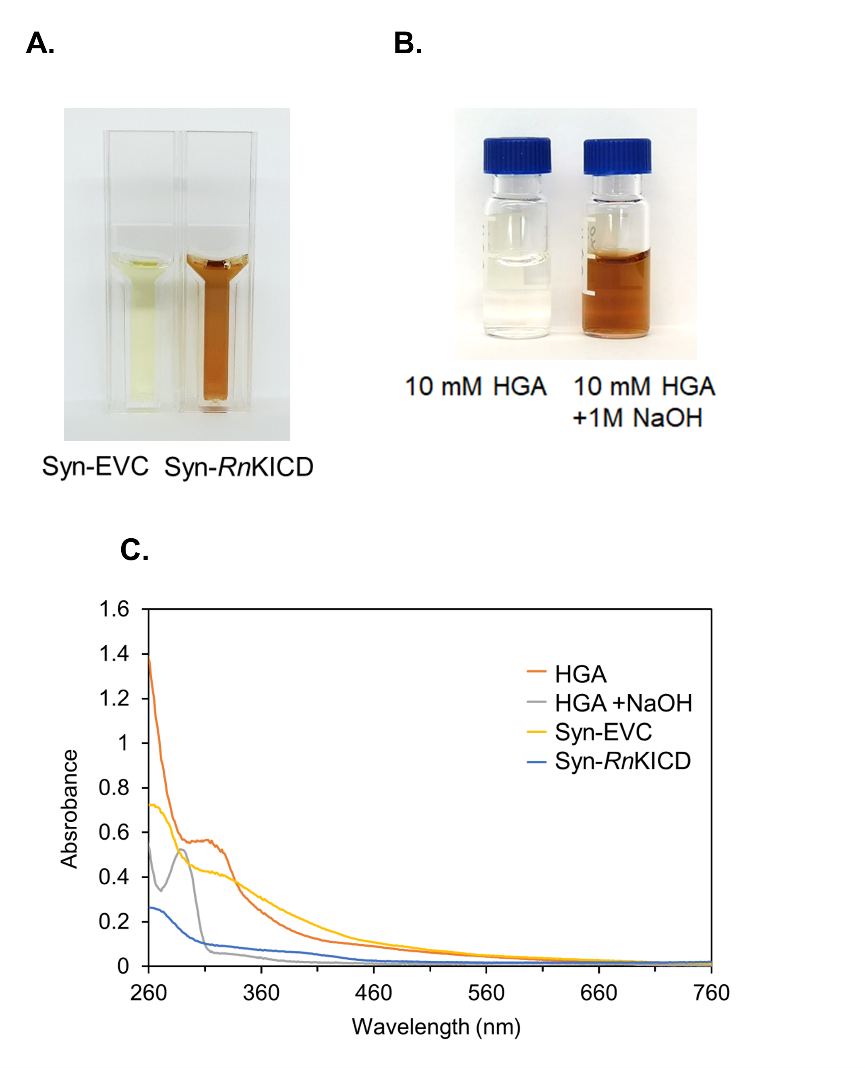
**

**Fig. S3.** Pyomelanin formation in high density cultivation (HDC) system. (A) The supernatant of Syn-EVC and Syn-*Rn*KICD grown in HDC system. (B) 10 mM homogentisate (HGA) was dissolved in water and 10 mM HGA oxidized with 1 M NaOH for 1 h (C) UV-Vis absorption spectra of 0.2 mM HGA, 0.2 mM HGA oxidized with NaOH, supernatant of Syn-EVC and Syn-*Rn*KICD. Supernatant of Syn-EVC and Syn-*Rn*KICD from high density cultivation was diluted 50 times for the absorbance measurement.

**Table S1.** Codon optimized genes used in this study. The gene sequences were codon optimized for expression in *Synechocystis* sp. PCC 6803 with Gene Designer 2.0 software. The genes synthesized by GenScript was delivered in pUC57 plasmids cloned into *Xba*I and *Pst*I sites. The *Bgl*II and *Spe*I restrictions sites were added to the beginning and the end of the sequences, respectively, to further facilitate the subcloning.

| **Synthesized genes** | **Sequence (5’-3’)** |
| --- | --- |
| *Rn*KICD | ATGACTACCTATTCCAACAAGGGACCAAAACCAGAACGGGGGCGTTTTCTCCACTTTCATTCCGTGACTTTTTGGGTTGGTAATGCCAAGCAGGCCGCGAGCTTCTATTGCAACAAAATGGGCTTTGAACCCTTAGCTTATAAAGGCTTGGAAACTGGTAGCCGCGAAGTGGTGAGTCACGTTATCAAACAAGGAAAGATCGTCTTTGTTCTCTGTTCTGCCTTGAATCCCTGGAATAAAGAAATGGGTGATCATCTCGTAAAACACGGAGATGGAGTTAAAGACATTGCCTTCGAAGTGGAAGACTGCGAACATATTGTCCAAAAGGCGCGTGAACGCGGTGCGAAAATTGTTCGAGAGCCATGGGTGGAAGAGGACAAGTTTGGAAAAGTAAAATTTGCCGTGCTTCAAACTTACGGCGATACCACCCATACCCTCGTAGAAAAAATTAATTACACTGGGCGATTTCTGCCGGGCTTTGAAGCCCCCACCTATAAGGATACTTTATTACCCAAGTTGCCATCTTGTAATTTAGAAATTATTGACCATATTGTGGGTAATCAGCCAGATCAGGAAATGGAATCCGCGAGTGAGTGGTACTTAAAAAATTTACAGTTCCATCGGTTTTGGAGTGTGGATGATACCCAGGTGCATACCGAGTACAGTAGTCTCAGGAGCATTGTTGTGGCGAATTATGAAGAATCCATCAAAATGCCGATTAATGAGCCTGCTCCTGGCCGGAAAAAATCCCAAATTCAAGAGTATGTTGATTATAACGGTGGCGCCGGCGTACAGCATATCGCCCTGCGTACCGAAGATATTATTACCACTATTCGCCATCTGCGCGAACGCGGCATGGAATTTTTAGCCGTTCCAAGCAGTTATTATCGTCTCCTACGTGAAAACTTAAAAACTTCCAAGATCCAAGTGAAGGAGAATATGGATGTTCTCGAAGAATTAAAAATCCTTGTGGATTATGATGAAAAGGGCTATCTGCTGCAAATCTTTACTAAACCCATGCAGGACCGGCCCACTTTGTTTTTAGAGGTGATCCAGCGTCATAATCACCAAGGATTTGGCGCCGGCAATTTTAATTCCTTATTCAAGGCCTTTGAGGAAGAACAAGCCTTACGTGGCAATTTGACTGACTTAGAGACCAACGGCGTGAGATCCGGTATGTAA |
| *Pt*M3K | ATGGAGAATTACAATGTCAAGACCAGGGCGTTCCCAACCATCGGTATTATTTTATTGGGCGGTATTAGCGACAAAAAAAATCGGATTCCCTTACACACTACTGCTGGAATTGCCTACACTGGTATCAATAATGACGTTTATACTGAAACCAAATTATATGTCTCCAAAGATGAAAAATGCTATATTGATGGGAAAGAAATTGATCTCAATAGTGATAGGTCCCCCAGTAAAGTCATTGACAAGTTCAAGCATGAGATTCTCATGCGCGTAAATTTGGATGATGAGAACAATTTGAGTATTGATTCTCGGAACTTTAATATCTTGTCTGGGAGTAGCGATTCTGGTGCCGCTGCCCTAGGCGAATGCATCGAAAGTATTTTTGAGTATAACATCAACATCTTTACCTTTGAAAATGACCTACAACGTATTTCCGAAAGCGTCGGCCGTAGTCTCTATGGAGGTCTCACTGTTAACTACGCTAATGGACGCGAATCCCTAACTGAACCTCTGTTAGAACCTGAAGCCTTCAATAATTTTACCATCATTGGCGCTCATTTCAACATTGACCGAAAGCCCAGCAACGAGATTCACGAAAATATTATTAAACATGAGAATTACCGTGAACGTATTAAGTCCGCCGAGCGGAAGGCGAAGAAATTAGAAGAACTGAGTCGTAATGCCAATATCAAAGGTATTTTCGAACTGGCCGAGTCCGATACCGTGGAATACCACAAAATGTTGCATGATGTCGGAGTTGATATTATCAACGATCGAATGGAGAACCTCATTGAACGCGTGAAAGAAATGAAGAATAATTTTTGGAACTCTTATATTGTGACTGGTGGCCCCAACGTGTTTGTTATCACCGAAAAAAAGGACGTAGATAAAGCCATGGAGGGGCTTAACGATTTGTGTGATGATATTCGGTTGTTGAAAGTTGCCGGCAAACCACAAGTAATTAGTAAAAATTTTTAA |

**Table S2.** Oligonucleotides used in this study. [PHO] is 5' phosphate, restriction sites are in bold, RBS* is in small letters, strep-tag II is underlined.

| **Primer** | **Primer sequence (5’–3’)** | **Target gene** |
| --- | --- | --- |
| **For expression in pEEK2-*Pt*M3K-*Rn*KICD** | |  |
| F_M3K_BglII | TCAC**AGATCT**ATGGAGAATTACAATG | *ptm3k* |
| R_M3K_RBSs_SpeI | GCAT**ACTAGT**acctccactaTCAGAGTTCTTAAAAATTTTTACTAATTACTTGTGG | *ptm3k* |
| F_KICD_SpeI_LST | AT**ACTAGT**ATGTGGAGCCATCCTCAATTTGAAAAGGGCAGTGGTTCAGGTAGTATGACTACCTATTCCAACAA | *rnkicD* |
| R_KICD_SacII | ATCG**CCGCGG**TTACATACCGGATCTCACG | *rnkicD* |
| **For expression in pEEK2-*Pt*M3K-*Rn*KICD-F** | |  |
| F_fusion_MK3 | [PHO] GGCAGTGGTTCAGGTAGTATGA | *ptm3k* |
| R_fusion_MK3 | [PHO] AAAATTTTTACTAATTACTTGTGGTTTGCC | *ptm3k* |
